# Semiological differences between children and adults with temporal lobe epilepsy: a video-EEG based multivariate analysis

**DOI:** 10.3389/fneur.2025.1578958

**Published:** 2025-05-08

**Authors:** Aoxue Mei, Ke Xu, Mingkun Gong, Cong Fu, Yujie Bo, Jiao Qiao, Tianfu Li, Mengyang Wang, Xiongfei Wang, Jian Zhou, Yuguang Guan, Guoming Luan

**Affiliations:** ^1^Department of Neurosurgery, Sanbo Brain Hospital, Capital Medical University, Beijing, China; ^2^Department of Neurology, Sanbo Brain Hospital, Capital Medical University, Beijing, China; ^3^Laboratory for Clinical Medicine, Capital Medical University, Beijing, China

**Keywords:** temporal lobe epilepsy, seizure semiology, children and adults, multivariate analysis, anterior temporal lobectomy

## Abstract

**Objective:**

This study investigated the differences in semiological characteristics of temporal lobe epilepsy (TLE) between children and adult populations, specifically examining the impact of age on these manifestations. Using multivariate analysis, this study assessed the influence of age on TLE clinical manifestations, including seizure complexity, while controlling for etiology and lesion location.

**Methods:**

This study retrospectively analyzed clinical data from patients who underwent anterior temporal lobectomy (ATL) and achieved seizure-free at Sanbo Brain Hospital. Semiological features were analyzed using video-electroencephalogram (video-EEG) recordings. Following descriptive analysis of clinical characteristics and seizure symptom differences between children and adult cohorts, univariate and multivariate logistic regression analyses were performed to evaluate associations between clinical variables and specific seizure components.

**Results:**

A total of 176 patients (39 children and 137 adults) who underwent ATL and achieved seizure-free status met the inclusion criteria for this study. Significant differences were observed between children and adults in the incidence of: auras (*p = 0.023*), motor seizures (*p = 0.002*), clonic seizures (*p = 0.002*), focal to bilateral tonic–clonic seizures (*p = 0.028*), and lateralizing signs (*p = 0.038*). The incidence of automotor seizures (*OR = 1.05, 95% CI 1.00–1.09, p = 0.039*) and clonic seizures (*OR = 1.06, 95% CI 1.01–1.12, p = 0.039*) showed a positive correlation with increasing age.

**Significance:**

This study demonstrates significant age-dependent differences in semiological manifestations of TLE, suggesting that age-related neurodevelopmental changes underlie distinct seizure patterns. These findings support age-specific treatment strategies, as age affects TLE seizure patterns and clinical management decisions.

## Highlights

- Analysis of seizure symptom components through video-EEG recordings can effectively reduce recall bias and inaccurate symptom descriptions, providing more objective and reliable data.
- Significant differences were observed in the incidence of auras, motor seizures, clonic seizures, focal to bilateral tonic–clonic seizures, and lateralizing signs between children and adult TLE patients.
- Automotor and clonic seizure incidence exhibited an age-dependent increase.
- Multivariate regression analysis revealed age-related differences in seizure symptomatology, suggesting that brain maturation may contribute to the increased complexity of seizures in adults.
- Future longitudinal studies with larger cohorts and more diverse clinical backgrounds are needed to further verify the effect of age on seizure semiology and better understand underlying mechanisms.

## 1 Introduction

Temporal lobe epilepsy (TLE) represents the most common type of focal epilepsy. Approximately two-thirds of drug-resistant epilepsy cases across all age groups are diagnosed with TLE (1–3). Surgical intervention remains the primary treatment modality for medically refractory TLE, while accurate presurgical evaluation is crucial for optimizing surgical planning and postoperative outcomes. Notably, therapeutic responses and long-term outcomes exhibit marked differences between children and adult TLE populations (4, 5).

Seizure semiology, particularly the characteristics of seizure manifestations, plays a crucial role in the lateralization and localization of the epileptogenic zone (EZ). While existing studies have developed classification criteria for seizure symptoms, the majority have focused on individual symptoms or limited semiological features, neglecting the complex interactions among age, etiology, lesion location, and other clinical relevant factors. Furthermore, previous studies (6, 7) have indicated that the semiology of TLE evolves with age; however, the precise influence of age on seizure manifestations remains insufficiently characterized. This knowledge gap is partly due to methodological limitations in earlier studies, including restricted cohort selection, coarse seizure classification, and reliance on descriptive or univariate statistical analyses. In particular, seizures were often categorized broadly as motor or non-motor, without detailed analysis of specific subtypes, and key clinical variables, such as lesion type, lateralization, and disease duration, were frequently omitted.

To address these gaps, this study systematically investigates how age influences the semiological features of TLE, using multivariate logistic regression to assess the combined impact of age, etiology, and other clinical factors. Seizure semiology was evaluated through video-electroencephalogram (video-EEG) recordings, minimizing recall bias and enhancing the objectivity of symptom characterization. By providing a nuanced understanding of age-related variations in seizure semiology, this study may assist clinicians in refining preoperative assessments and informing age-appropriate treatment strategies in TLE.

## 2 Patients and methods

### 2.1 Patient selection

**Figure 1:**
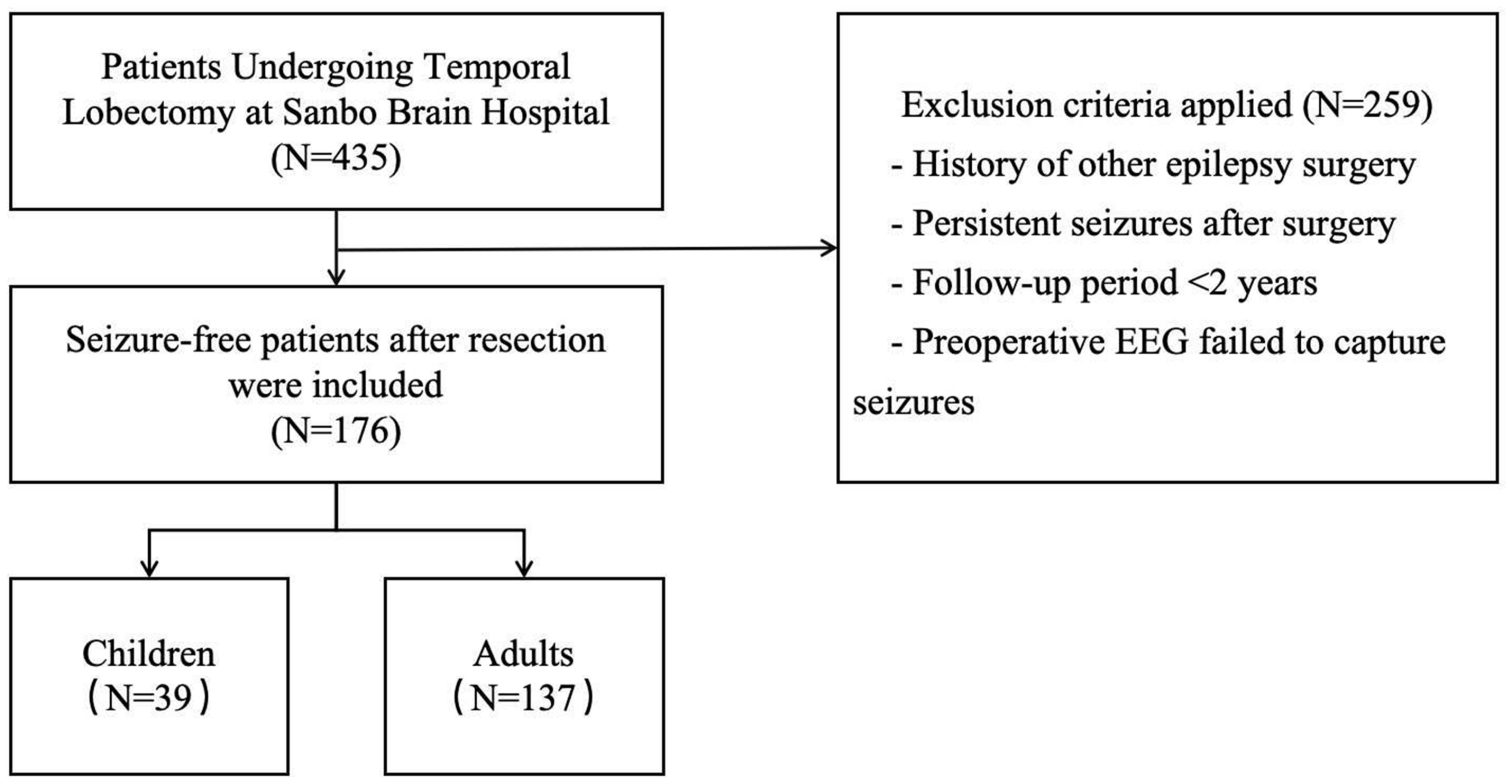
Outline of the study design and patient selection.

Clinical data were retrospectively extracted from electronic medical records of epilepsy patients managed at the Epilepsy Center of Sanbo Brain Hospital, Capital Medical University. All included patients had a minimum postoperative follow-up period of two years. Inclusion criteria comprised: (1) completion of comprehensive preoperative multidisciplinary evaluation, and (2) achievement of postoperative seizure freedom (Engel class I) following anterior temporal lobectomy (ATL). Exclusion criteria consisted of patients lacking definitive ictal recordings on preoperative scalp video-EEG monitoring. Participants were stratified by age into two cohorts: adults (≥18 years) and children (<18 years). Figure 1 summarizes the study design and patient selection workflow.

### 2.2 Seizure classification and video-EEG assessment

Seizure assessment and component analysis were based on preoperative video-EEG recordings. All patients underwent continuous video-EEG monitoring for a minimum of 24 h to optimize ictal event capture. Seizures were classified according to the four-dimensional epilepsy classification system proposed by Lüders et al. (8), which includes symptom-based categories such as simple motor, complex motor, and dyscognitive seizures. While these terms are not fully aligned with the ILAE 2017 classification (9), they were used in this study to capture detailed clinical semiology. For consistency, corresponding ILAE 2017 terms are referenced where applicable. Lateralizing signs were analyzed separately as clinical markers, not as seizure types.

Seizure symptom components were categorized as follows:

1. Auras (self-reported)
2. Autonomic seizures (tachycardic and bradycardic seizures)
3. Dyscognitive seizures (dialeptic and akinetic seizure)
4. Simple motor seizures (tonic, clonic, and versive seizure)
5. Complex motor seizure (automotor and hypermotor seizure)
6. Focal to bilateral tonic–clonic seizures (FBTCS)
7. Lateralizing signs [unilateral tonic and clonic seizure, unilateral limb hypermotor, unilateral blinking (10–13)]

Seizure semiology was independently evaluated by two board-certified epileptologists. Auras were specifically documented through structured patient interviews, with all subjective reports subsequently verified by attending neurologists during comprehensive clinical evaluations. Classification discrepancies were resolved through consensus review by a senior epileptologist with over 15 years of experience, whose judgment was considered the gold standard.

### 2.3 Surgical procedures and histopathological classification

All patients underwent standard ATL for resection of the epileptogenic zone. A 3.0–3.5 cm segment of the anterolateral temporal cortex was resected in dominant hemisphere cases, whereas 4.0–4.5 cm was removed in non-dominant hemisphere procedures.

Mesial structures, including the amygdala and the anterior 3.0 cm of the hippocampus, were excised en bloc, following the surgical technique described by Spencer et al. (14). For patients with temporal lobe tumors, ATL was combined with lesionectomy.

Postoperative histopathological diagnoses were categorized as follows (15): isolated hippocampal sclerosis (HS), isolated focal cortical dysplasia (FCD), FCD type IIIa (FCD with HS), FCD type IIIb (FCD with tumor), and isolated low-grade tumors (e.g., ganglioglioma, DNET). All surgical specimens were classified according to the ILAE guidelines for FCD and WHO tumor grading standards.

### 2.4 Postoperative follow-up

Postoperative follow-up assessments were conducted at 3 months and annually thereafter, including routine 16-h scalp video-EEG and magnetic resonance imaging (MRI). Complications such as intracranial hemorrhage, central nervous system infection, and neurological deficits were documented. Seizure recurrence was defined as any postoperative seizure occurring beyond the first postoperative week. Surgical outcomes were classified using the Engel classification system (16), with Engel Class I outcomes during the most recent two years considered favorable, and Classes II–IV deemed unfavorable.

### 2.5 Statistical analysis

Descriptive statistics were used to summarize the demographic and clinical characteristics of the study population. Continuous variables with normal distribution were presented as mean ± standard deviation (SD), while non-normally distributed variables were reported as median (interquartile range [IQR]). Categorical variables were expressed as counts (percentages). Between-group comparisons of categorical variables were conducted using either Pearson’s chi-square test or Fisher’s exact test, depending on expected cell frequencies. Nonparametric analyses (Mann–Whitney U test for two-group comparisons; Kruskal-Wallis test for multiple groups) were employed to assess age distributions across seizure status groups. To evaluate potential predictors of specific seizure semiology, we performed both univariate and multivariate logistic regression analyses. All analyses were conducted using SPSS Statistics version 25.0 (IBM Corp., Armonk, NY), with statistical significance set at *α* = 0.05 (two-tailed).

## 3 Results

Among 290 consecutive patients who underwent ATL between 2008 and 2020, 190 (65.5%) achieved sustained seizure free (Engel Class I) for at least two years postoperatively. 14 patients were excluded due to the absence of definitive ictal events on preoperative scalp video-EEG monitoring. The final study cohort included 176 patients who met all inclusion criteria, comprising 39 children (<18 years) and 137 adults (≥18 years). Demographic and clinical characteristics of the cohort are summarized in Table 1.

**Table 1:** The demographic characteristics of temporal lobe epilepsy children and adults.

Variables/Characteristics	Children *N* = 39	Adults *N* = 137
Gender: male vs. female	22 (56.4%):17(43.6%)	69 (50.4%):68(49.6%)
Side of surgery: left vs. right	19 (48.7%):20(51.3%)	72 (52.6%):65(47.4%)
Median seizure duration at surgery: years (range)	5.0 (1.0–18.0)	15.0 (1.0–53.0)
Median age at surgery: years (range)	11.8 (2.6–17.8)	25.7 (18.4–61.3)
Positive MRI results	35 (89.7%)	128 (93.4%)
Personal history		
Febrile seizures	6 (15.4%)	37 (27.0%)
Hypoxic ischemic encephalopathy	5 (12.8%)	4 (2.9%)
Encephalitis	2 (5.1%)	12 (8.8%)
Cerebral trauma	2 (5.1%)	8 (5.8%)
Interictal video-EEG		
Focal	24 (61.5%)	90 (65.7%)
Unilateral	10 (25.7%)	10 (7.3%)
Multiple foci	3 (7.7%)	34 (24.8%)
Generalized	2 (5.1%)	1 (0.7%)
No epileptiform discharges	0	2 (1.5%)
Ictal video-EEG		
Total number of recorded seizures	227	456
Median number of recorded seizures (range)	4 (1–43)	3 (1–9)
Focal	15 (38.5%)	64 (46.7%)
Unilateral	15 (38.5%)	46 (33.6%)
Multiple foci	1 (2.6%)	9 (6.6%)
Generalized	8 (20.5%)	17 (12.4%)
Neuropathology		
Isolated HS	1 (2.6%)	12 (8.8%)
Isolated FCD	8(20.5%)	14 (10.2%)
FCD IIIa (FCD with HS)	9(23.1%)	90 (65.7%)
FCD IIIb (FCD with tumor)	1(2.6%)	2(1.5%)
Isolated Low-Grade Tumors	14 (35.9%)	11 (8.0%)

HS: Hippocampal sclerosis, FCD: Focal cortical dysplasia.

The surgical cohort included 176 patients, with 91 (51.7%) undergoing left-sided and 85 (48.3%) undergoing right-sided ATL. Preoperative MRI abnormalities were detected in 163 patients (92.6%). The most common histopathological diagnoses were focal cortical dysplasia (FCD) in 124 patients (70.5%) and hippocampal sclerosis (HS) in 117 patients (66.5%). Scalp video-EEG monitoring recorded a total of 683 seizures, with a median of 3 seizures per patient (range: 1–43). The median age at preoperative video-EEG evaluation was 24.5 years (range: 2.5–61.3 years).

Autonomic seizures featuring ictal tachycardia (heart rate increase of 30–60 bpm) were observed in 79 patients (44.9%), as recorded by continuous cardiac monitoring. Dialeptic seizures were documented in 25 patients (14.2%) and akinetic seizures in 48 (27.3%). Simple motor seizures included clonic (*n* = 101, 57.4%), tonic (*n* = 21, 11.9%), and versive (*n* = 25, 14.2%) presentations. Complex motor seizures were characterized by automotor (*n* = 125, 71.0%) and hypermotor (*n* = 45, 25.6%) manifestations. FBTCS were identified in 53 patients (30.1%), while auras were reported by 72 patients (40.9%). The frequency distribution of these semiological components is summarized in Table 2.

**Table 2:** Descriptive statistics of temporal lobe epilepsy semiology.

Semiology variables	Total number	N (frequency)			Median age (25–75% range)		
		Children group (*N* = 39)	Adult group (*N* = 137)	*p* value (children vs. adult)	Group with specific seizure	Group without specific seizure	*p* value
Aura	72 (40.9%)	10 (25.6%)	62 (45.3%)	**0.028**	24.4 (20.2–29.5)	24.2 (15.7–28.5)	0.308
Autonomic seizure	79 (44.9%)	17 (43.6%)	62 (45.3%)	0.854	23.2 (18.6–26.9)	25.4 (19.6–31.1)	0.081
Dialeptic seizure	25 (14.2%)	4 (10.3%)	21 (15.3%)	0.423	24.6 (20.2–27.1)	24.1 (18.6–28.9)	0.954
Akinetic seizure	48 (27.3%)	7 (17.9%)	41 (29.9%)	0.098	25.8 (22.5–30.8)	23.9 (17.9–28.3)	0.124
Tonic seizure	101 (57.4%)	19 (48.7%)	82 (59.9%)	0.215	24.0 (20.3–29.0)	24.5 (16.2–28.9)	0.448
Clonic seizure	21 (11.9%)	1(2.6%)	20 (14.6%)	0.041*	26.6 (23.8–36.9)	23.7 (18.4–28.5)	**0.007**
Versive seizure	25 (14.2%)	2 (5.1%)	23 (16.8%)	0.066*	25.8 (22.2–29.0)	24.0 (18.4–28.9)	0.108
Automotor seizure	125 (71%)	20 (51.3%)	105 (76.6%)	**0.002**	25.2 (20.6–29.2)	21.7 (11.8–27.4)	**0.005**
Hypermotor seizure	45 (25.6%)	7 (17.9%)	38 (27.7%)	0.216	25.2 (18.9–29.6)	24.0 (18.8–28.4)	0.517
FBTCS	53 (30.1%)	6 (15.4%)	47 (34.3%)	**0.023**	24.7 (21.5–28.0)	23.5 (15.7–29.1)	0.171
Lateralizing signs	89 (50.6%)	14 (35.9%)	75 (54.7%)	**0.038**	23.7 (20.3–26.8)	25.6 (14.9–30.0)	0.548

*Fisher’s precision probability test. The left part of the table compares the main seizure symptom components of temporal lobe epilepsy across the total number, children, and adult groups. The right part of the table compares the median age of patients with or without certain seizure symptom components. Bold values indicate statistically significant *p*-values (*p* < 0.05).

**Figure 2:**
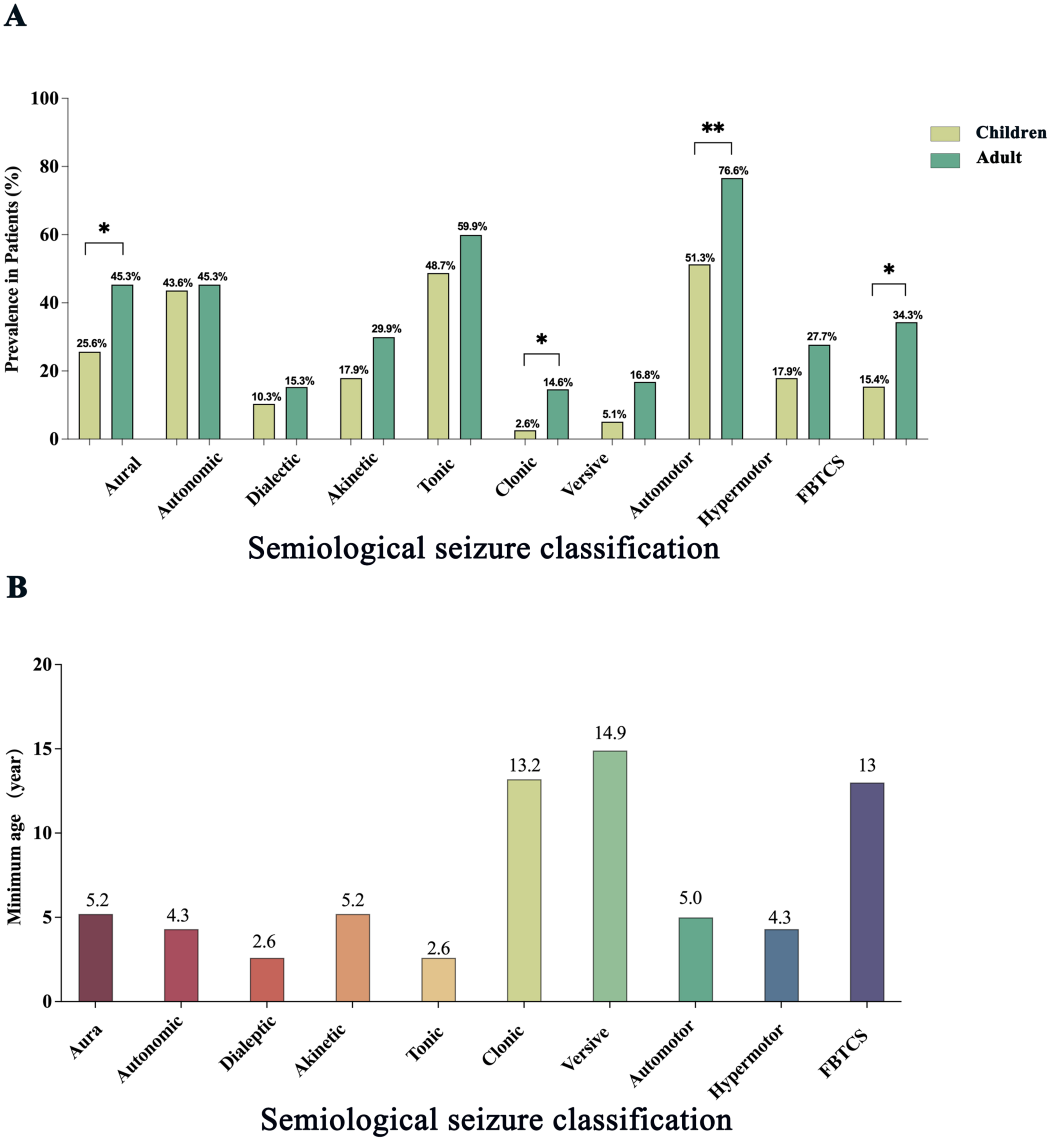
Age-related differences in seizure symptom prevalence and onset in patients with temporal lobe epilepsy. **(A)** Comparison of the prevalence of major seizure symptom components between children and adults with temporal lobe epilepsy. Bars indicate the proportion of patients exhibiting each symptom type in the two groups, with children shown in pale green and adults in dark green. Statistically significant differences are indicated by asterisks (*p < 0.05; **p < 0.01). **(B)** Minimum age at which different seizure symptom components were observed. Bars represent the youngest age at which each symptom type was recorded across all patients.

Age-related differences in seizure semiology between children and adult TLE patients are detailed in Table 2 and illustrated in Figure 2A. Significant intergroup differences were observed in auras (*p* = 0.023), automotor seizures (*p = 0.002*), clonic seizures (*p = 0.002*), and FBTCS (*p = 0.028*). Lateralizing signs demonstrated significantly greater prevalence among adults compared to children (*p = 0.038*). Further comparison of median ages between patients with and without specific seizure types revealed significant differences for clonic (*p = 0.007*) and automotor (*p = 0.005*) seizures. The earliest age at onset for each seizure type is illustrated in Figure 2B. Dialeptic and tonic seizures were observed as early as 2.6 years of age. In contrast, versive and clonic seizures were not observed until later childhood, with the youngest cases reported at 14.9 and 13.2 years, respectively.

Based on the descriptive findings, univariate and multivariate logistic regression analyses were performed to explore associations between age and the occurrence of auras, automotor seizures, clonic seizures, and FBTCS. Advancing age was significantly associated with higher odds of automotor seizures (OR = 1.05, 95% CI: 1.00–1.09, *p* = 0.039) and clonic seizures (OR = 1.05, 95% CI: 1.00–1.09, *p* = 0.039). Age at the time of video-EEG monitoring emerged as an independent predictor of these seizure types. No other clinical variables demonstrated statistically significant associations. Full model results are provided in Tables 3, 4.

**Table 3:** Univariate and multivariate logistic analysis of automotor seizure in patients with temporal lobe epilepsy.

	Univariate analysis			Multivariate analysis		
	OR	95% CI	*p* value	OR	95% CI	*p* value
Male gender	1.2	0.62–2.30	0.588	-		
Left side of surgery	0.96	0.50–1.84	0.902	-		
Age at EEG recording	1.05	1.02–1.10	0.004	1.05	1.00–1.09	**0.039**
Duration of seizure	1.04	0.99–1.08	0.078	1.00	0.95–1.05	0.964
Focal cortical dysplasia	0.41	0.21–0.83	0.013	0.49	0.22–1.09	0.081
Hippocampal sclerosis	0.49	0.25–0.97	0.039	0.83	0.36–1.90	0.662
Positive MRI results	2.36	0.51–11.07	0.275	-		
Febrile seizure history	0.57	0.25–1.30	0.574	-		

Bold values indicate statistically significant *p*-values (*p* < 0.05).

**Table 4:** Univariate and multivariate logistic analysis of clonic seizure in patients with temporal lobe epilepsy.

	Univariate analysis			Multivariate analysis		
	OR	95% CI	*p* value	OR	95% CI	*p* value
Male gender	0.83	0.33–2.07	0.690	-		
Left side of surgery	1.28	0.51–3.22	0.596	-		
Age at EEG recording	1.07	1.02–1.12	0.006	1.06	1.01–1.12	**0.039**
Duration of seizure	1.06	1.01–1.11	0.022	1.02	0.97–1.08	0.396
Focal cortical dysplasia	0.53	0.17–1.64	0.268	0.76	0.21–2.70	0.670
Hippocampal sclerosis	2.33	0.75–7.29	0.144	0.66	0.18–2.35	0.517
Positive MRI results	0.60	0.07–4.83	0.628	-		
Febrile seizure history	1.43	0.45–4.50	0.542	-		

Bold values indicate statistically significant *p*-values (*p* < 0.05).

## 4 Discussion

This study systematically investigated age-related differences in the semiological features of TLE using multivariate logistic regression analysis. The regression models incorporated FCD and hippocampal HS as key variables, representing neocortical and medial temporal epileptogenic pathologies, respectively. While the influence of lesion type and location on seizure semiology is well established, the effect of age on ictal manifestations remains comparatively underexplored. To ensure accuracy and objectivity, semiological features were classified based on preoperative scalp video-EEG recordings, with all events assessed through direct video review by trained epileptologists rather than relying on patient or caregiver reports. This methodological approach reduced recall bias and enhanced the reliability of seizure classification by enabling standardized evaluation of ictal patterns.

### 4.1 Age-dependent patterns of motor seizures

In this study, adults with TLE exhibited significantly higher rates of automotor seizures, clonic seizures, tonic seizures, versive seizures, and FBTCS compared to children, with a positive correlation between age and the incidence of tonic seizures. These findings suggest that age contributes to the complexity and diversity of motor seizure semiology in TLE patients (17). This pattern is consistent with prior studies indicating that maturation of cortical circuits enables more extensive neural network involvement during seizures (18, 19). In adults, the EZ often expands to multiple cortical areas, resulting in more complex seizure manifestations. In contrast, immature neural networks in young children, particularly those of preschool age, limit the range and complexity of motor seizure symptoms. Notably, the youngest patient exhibiting automotor seizures in this cohort was five years old, consistent with reports of low automotor seizure frequency in early childhood, likely due to underdeveloped motor functions such as chewing or hand movements (20–23). Similarly, FBTCS was first observed at age 13, aligning with evidence that bilateral seizure propagation requires cortical maturation, dendritic growth, myelination, and interhemispheric synchronization, which are incomplete in early childhood (24, 25).

Contrary to our findings, two earlier studies reported an inverse relationship between age and motor seizure frequency, suggesting that motor manifestations decrease with age (6, 7). While prior studies have contributed to the classification of seizure types, most have focused on isolated symptoms or small subsets of semiological features, often neglecting the interplay of age, etiology, lesion location, and other clinical variables in shaping seizure presentations. Evidence suggests that TLE semiology evolves with age, particularly in relation to auras, automatisms, and motor seizures (6, 7). However, these investigations primarily employed descriptive analyses and univariate statistical methods, which limited their ability to control for confounding variables and to accurately quantify the specific impact of age on seizure semiology. Notably, the 2002 study (6) included only 15 children under six years of age, and seizure manifestations were classified into broad motor and non-motor categories, failing to capture the full complexity and diversity of TLE symptomatology. As an exploratory analysis, its findings had limited generalizability. Although the 2007 study (7) expanded the age range to include adults, it still suffered from a relatively small sample size and continued reliance on descriptive statistics. Moreover, it did not account for key clinical variables such as lesion type, lateralization, or seizure duration, nor did it employ multivariate models to isolate the effect of age from other potential influences. In contrast, the present study conducted a systematic, age-stratified comparison between children and adults with TLE, encompassing a larger and more diverse cohort (ages 2.5 to 61 years). Utilizing the comprehensive four-dimensional classification framework proposed by Lüders et al. (8), seizure components were rigorously categorized and analyzed. By applying multivariate logistic regression, this study examined the combined effects of age, etiology (e.g., hippocampal sclerosis, focal cortical dysplasia), and other clinical variables on seizure semiology. All seizure features were evaluated using video-EEG recordings, thereby minimizing recall bias and enhancing data objectivity. These analyses aim to provide a more nuanced understanding of how age and associated neurodevelopmental changes may influence seizure semiology in TLE. By addressing the limitations of previous studies and incorporating a broader set of clinical variables, this study seeks to offer preliminary insights that could inform future research and contribute to the refinement of individualized diagnostic and treatment strategies in epilepsy care.

### 4.2 Influence of epilepsy duration on seizure semiology

In addition to age, disease duration may independently influence the semiological features of the TLE. In this study cohort, adult patients exhibited a significantly longer epilepsy duration prior to surgery (median: 15 years) compared to children (median: 5 years). This longer disease course may contribute to the higher incidence of complex seizure types observed in adults, including automotor seizures (76.6% vs. 51.3%), clonic seizures (14.6% vs. 2.6%), and FBTCS (34.3% vs. 15.4%). Additionally, lateralizing signs were more frequently observed in adults (54.7% vs. 35.9%), suggesting broader cortical involvement. Previous studies (26) have shown that long-standing epilepsy is associated with seizure type diversification and increased bilateral propagation, particularly in TLE. Chronic epileptic activity may induce network remodeling, characterized by functional and structural alterations that facilitate the spread of epileptiform discharges beyond the initial EZ (27). Such changes may contribute to the emergence of more complex seizure patterns, including FBTCS, complex motor seizures, and more prominent lateralizing signs.

Notably, while multivariate analysis in this study identified age as an independent predictor for automotor and clonic seizures, epilepsy duration showed a positive correlation with clonic seizures in univariate analysis (OR = 1.06, *p* = 0.022), though it was not significant in multivariate modeling (*p* = 0.396). Given the close relationship between age and disease duration in adults, it is likely that both factors jointly contribute to the increased complexity of seizure manifestations in long-standing TLE. The presence of FBTCS or frequent complex motor seizures may indicate a longer epilepsy history, aiding clinicians in estimating disease progression and tailoring treatment strategies.

In addition to seizure type diversification, we also observed a higher incidence of lateralizing signs in adults (54.7%) compared to children (35.9%). Although previous studies have reported a general absence of lateralizing signs in young children (28), the role of age in this context remains controversial, with inconsistent findings across studies (7, 20). These results suggest that longer epilepsy duration and brain maturation may jointly contribute to more prominent lateralizing signs in adults, which could assist in seizure localization during presurgical evaluation.

### 4.3 Aura and its impact on TLE diagnosis

An aura is typically defined as the initial subjective manifestation of a focal seizure that occurs without impairment of consciousness (9). In TLE, abdominal, affective, and mnemonic auras are often regarded as important localizing signs (29–31). However, the presentation of auras appears to vary with age. Older children and adults are more likely to report complex auras, such as affective or mnemonic experiences, whereas younger children, particularly those of preschool age, typically exhibit simpler auras, including abdominal discomfort or sensory phenomena. Diagnosing auras in young children poses particular challenges, as their limited language and cognitive development may hinder accurate description of subjective sensations. This can complicate both the identification and classification of auras, potentially leading to under diagnosis or misinterpretation. Given these challenges, it is essential to corroborate reported auras with other clinical indicators or diagnostic tools, such as video-EEG or caregiver observations. In this study, although a statistically significant difference in aura occurrence was observed between children and adults, multivariate regression analysis did not reveal a significant association between age and aura occurrence, suggesting that confounding factors may have influenced this finding. Due to concerns regarding subjective reporting bias and sample size limitations, we did not further subclassify aura types in this cohort. Future studies with larger and more diverse populations may help clarify age-related patterns in aura presentation and their clinical utility for seizure localization.

### 4.4 Autonomic and dyscognitive seizures in TLE

Autonomic seizures and dyscognitive seizures are typical features of TLE semiology. In this study, no significant age-related differences were observed in the incidence of either seizure type, with comparable frequencies in adults and children. Autonomic seizures encompass a variety of symptoms, including tachycardia, bradycardia, abdominal discomfort, urinary urgency, and emesis. These seizures may provide valuable lateralizing or localizing information, particularly when accompanied by heart rate changes. In this cohort, 44.9% of patients with autonomic seizures exhibited tachycardia. These findings indicate that autonomic manifestations, especially those involving cardiac responses, may assist in identifying the EZ (32–34).

The accurate diagnosis of dyscognitive seizures, typically characterized by a 的reduction or cessation of voluntary movements, requires patient cooperation, which poses challenges in children populations. Although such behavioral changes can be captured on video-EEG, patient self-report is often necessary to confirm associated symptoms such as impaired awareness or short-term memory loss. In young children, particularly those under 3 years of age, these seizures frequently manifest as motionless staring, a non-specific sign that can be easily misinterpreted as interictal behavior (35–37).

### 4.5 Study design, methodological strengths, and limitations

This study included only patients who achieved at least two years of seizure freedom following ATL to ensure a definitive diagnosis of unilateral TLE. Previous research (38) has demonstrated that a two-year seizure-free period is a strong predictor of long-term surgical success, with approximately 90% of such patients remaining seizure-free for up to 16 years postoperatively. Similarly, Janszky et al. (39) reported that patients who were seizure-free at two years post-surgery typically had well-localized epileptogenic foci within the temporal lobe and were more likely to maintain long-term seizure remission. This inclusion criterion therefore increased the likelihood of a single, well-defined EZ, thereby enhancing the reliability of semiological analysis and outcome assessment.

**Figure 3:**
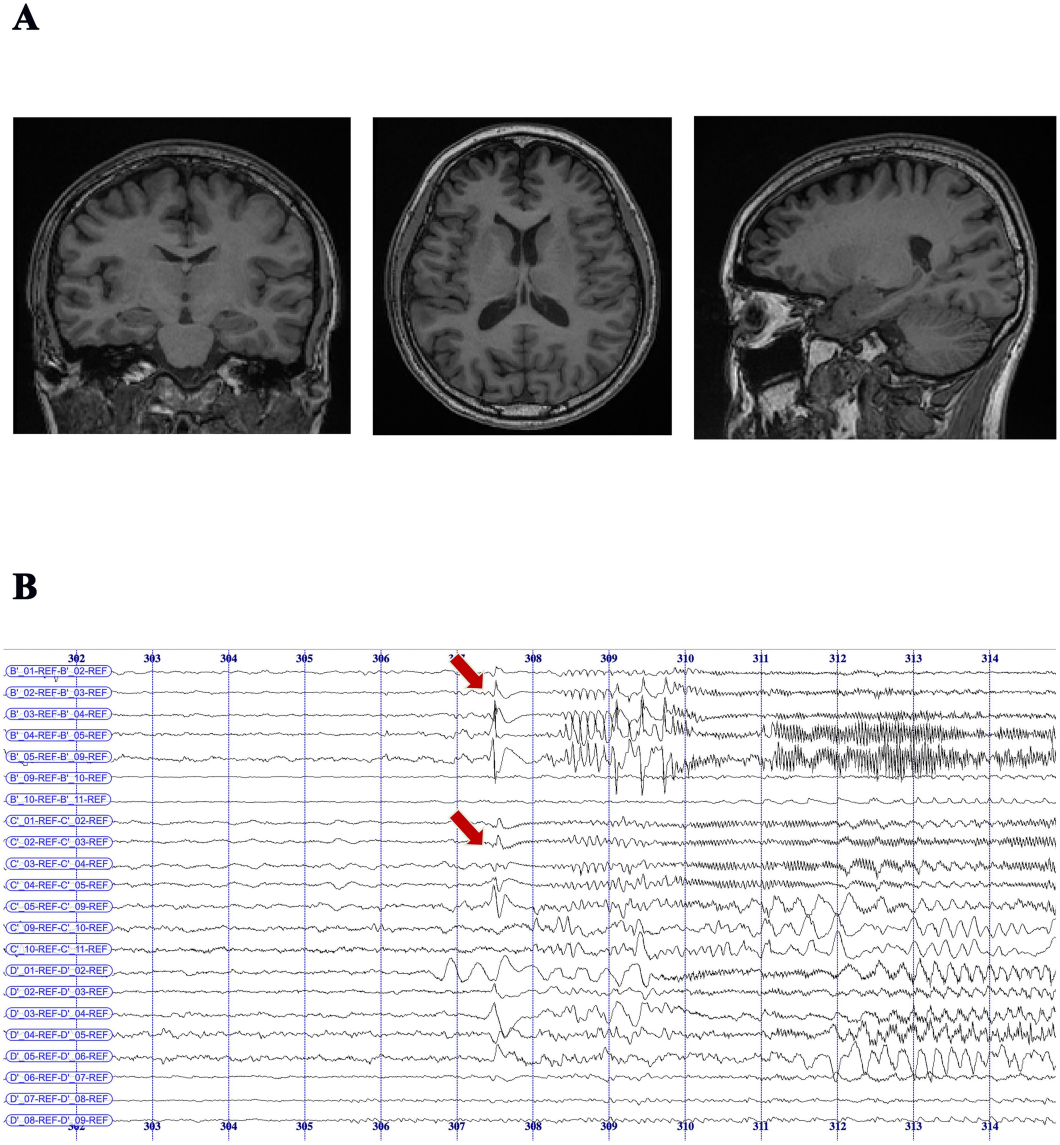
MRI-negative temporal lobe epilepsy patient with seizure onset localized by SEEG monitoring. **(A)** Preoperative MRI scans (coronal, axial, and sagittal T1-weighted images) showing no visible structural abnormalities in the bilateral temporal lobes, consistent with an MRI-negative diagnosis. **(B)** SEEG recording in an MRI-negative patient demonstrated seizure onset at B′ 1–4 (middle temporal gyrus–hippocampal head) and C′ 1–4 (middle temporal gyrus–hippocampal tail), characterized by initial spike-and-slow-wave discharges that evolved into either low-voltage fast activity or rhythmic 9–10 Hz spike-and-slow-wave complexes, followed by 11–12 Hz rhythmic fast activity at the same contacts. Recordings were acquired using 8–16 contact depth electrodes (2 mm length, 0.8 mm diameter, 1.5 mm spacing), implanted in the unilateral hippocampus and related regions based on presurgical evaluation. SEEG data were recorded with the Nicolet^™^ system (sampling rate: 512 Hz; bandpass filter: 0.1–200 Hz).

Accurate seizure localization is essential for effective surgical planning in drug-resistant TLE. However, the number of seizures captured during video-EEG monitoring is often limited, particularly in patients with low seizure frequency. In this study, the median number of recorded seizures per patient was 3 (range: 1–43); notably, 24 patients had only one clinical seizure recorded, with seizure intervals typically spanning two to four weeks. Similarly, among the 13 patients with MRI-negative TLE, 11 demonstrated consistent localization of seizure onset to the unilateral temporal lobe through non-invasive multimodal evaluation, including detailed seizure history, witness reports, high-resolution neuroimaging, interictal EEG, and neuropsychological testing. Despite limited ictal data or inconclusive MRI findings, the convergence of clinical, electrophysiological, and neuroimaging evidence supported the EZ hypothesis in all these patients. After multidisciplinary case discussion, surgical intervention was deemed appropriate per established criteria for drug-resistant TLE. This approach was particularly crucial in MRI-negative cases, where precise presurgical localization significantly influenced surgical candidacy and outcomes. Previous studies (40) have shown that up to 75% of MRI-negative TLE patients with concordant presurgical evaluations can achieve sustained seizure freedom. In the remaining two MRI-negative patients, non-invasive results were inconclusive, necessitating invasive EEG monitoring. For instance, in one representative case (Figure 3), despite a negative MRI (Figure 3A), stereo-EEG (SEEG) recordings localized seizure onset to mesial temporal structures, including the middle temporal gyrus and hippocampus (Figure 3B).

Despite these methodological strengths, this study has several limitations that should be acknowledged. First, as a single-center retrospective analysis, it is subject to recall bias. Second, due to the limited sample size, we were unable to analyze all typical semiological components of TLE or classify certain components, such as auras, into more detailed subtypes. Additionally, only patients who became seizure-free post-surgery were included to ensure diagnostic accuracy based on well-localized EZ. However, this criterion may limit generalizability, as it excludes patients with persistent seizures or those ineligible for surgery, who may exhibit more atypical or complex seizure patterns not represented in this cohort.

## 5 Conclusion

We observed significant differences in various seizure symptom components between children and adults with TLE. Multivariate regression analysis revealed an association between increasing age and a higher incidence of automotor and clonic seizures in adults with TLE. Brain maturation and accumulated life experiences may contribute to these changes in seizure symptomatology. This suggests that clinicians should exercise greater caution when evaluating adults suspected of having TLE, as the more complex presentation of symptoms could influence surgical outcomes. Given the complexity of these findings, further validation through prospective longitudinal studies or multi-center patient observations is necessary to confirm these results and better understand the underlying mechanisms.

## Data Availability

The datasets presented in this article are not readily available because the dataset used in this research contains sensitive clinical information related to patients with temporal lobe epilepsy (TLE). Access to the data is restricted to authorized researchers with approval from the ethics review board. All patient data has been anonymized to comply with data protection regulations. The dataset cannot be shared publicly due to confidentiality agreements and is subject to relevant ethical guidelines and local legislation. The data is available for use only within the context of the research as outlined in this study. Requests to access the datasets should be directed to Aoxue Mei, 18983894039@163.com.
